# A Signal-On Microelectrode Electrochemical Aptamer Sensor Based on AuNPs–MXene for Alpha-Fetoprotein Determination

**DOI:** 10.3390/s24247878

**Published:** 2024-12-10

**Authors:** Xiaoyu Su, Junbiao Chen, Shanshan Wu, Yong Qiu, Yuxiang Pan

**Affiliations:** 1 Innovation Platform of Micro/Nano Technology for Biosensing, ZJU-Hangzhou Global Scientific and Technological Innovation Center, Zhejiang University, Hangzhou 311200, China; 2 Hangzhou Shuntai Installation Engineering Co., Ltd., Hangzhou 311200, China; 3 Biosensor National Special Laboratory, Department of Biomedical Engineering, Zhejiang University, Hangzhou 310027, China; 4 Binjiang Institute, Zhejiang University, Hangzhou 310053, China

**Keywords:** alpha-fetoprotein, electrochemical sensor, aptamers, microelectrodes, AuNPs–MXene

## Abstract

As a crucial biomarker for the early warning and prognosis of liver cancer diseases, elevated levels of alpha-fetoprotein (AFP) are associated with hepatocellular carcinoma and germ cell tumors. Herein, we present a novel signal-on electrochemical aptamer sensor, utilizing AuNPs–MXene composite materials, for sensitive AFP quantitation. The AuNPs–MXene composite was synthesized through a simple one-step method and modified on portable microelectrodes. As signal molecules, AFP aptamers were conjugated with methylene blue (MB) and immobilized on the electrode surface. When interacting with AFP, conformational changes in the aptamer–target complex caused MB to approach the electrode, and the electrochemical signal was enhanced through signal-on mechanisms. The developed sensor demonstrated high sensitivity and selectivity for AFP, with a log-linear relationship defined as 1–300 ng/mL, and the LOD was 0.05 ng/mL (S/N = 3). The method was applied to laboratorial and real clinical samples and presented satisfactory selectivity, reproducibility, and long-term stability. The proposed high-performance sensor highlights the potential of electrochemical aptamer sensors in improving the warning capabilities in disease management.

## 1. Introduction

Alpha-fetoprotein (AFP), a primarily produced by the fetal liver and yolk sac, has normally low concentrations in healthy adults [1]. The levels of AFP exhibit significant changes in various pathological conditions, making it a widely used biomarker in clinical management [2]. In particular, AFP measurement is critical for the early warning and prognosis evaluation of liver cancer, hepatitis, and other liver diseases [3,4]. Elevated levels of AFP in adults are primarily associated with hepatocellular carcinoma and germ cell tumors, making it a crucial biomarker for the monitoring of these malignancies [5,6]. AFP levels are also utilized to screen high-risk populations, especially individuals with chronic liver diseases, such as hepatitis B and C infections or cirrhosis. In healthy human serum, the concentration of AFP is usually less than 25 ng/mL, and a high concentration of AFP may indicate a risk of disease. However, traditional AFP determination methods, including enzyme-linked immunosorbent assay and radioimmunoassay, are limited by sensitivity, specificity, and real-time determination capabilities [7,8,9]. Therefore, developing novel high-sensitivity and high-specificity AFP assay methods is of significant clinical importance.

Electrochemical sensors have obtained considerable attention in recent years due to their high sensitivity, rapid response, and simple operation in the field of biological determination [10,11]. Compared to conventional biosensor technologies, electrochemical sensors present notable advantages in sensitivity, selectivity [12]. The crucial point of an electrochemical sensor is the design of electrode materials and signal transduction mechanisms, which directly impact sensor performance [13]. Particularly, portable electrochemical microelectrodes (MEs) have gained significant attention in the field of APF determination due to their ability to provide real-time, sensitive, and selective determination of various analytes [14]. Typically, the ME is a miniaturized electrode that can be easily integrated into portable devices, enabling in situ measurements in clinical monitoring [15]. Compared to traditional macroelectrodes, the outstanding advantages of portable electrochemical MEs is that the mass transport characteristics are efficiently enhanced, which facilitates faster response times [16]. Furthermore, the size of ME allows for localized measurements and the integrated portability of the assay device [17,18].

Aptamers, a class of single-stranded nucleic acid molecules with specific binding capabilities, efficiently bind to target molecules [19,20]. Profiting from their excellent selectivity, stability, and synthesizability, aptamers have become critical recognition elements in biosensors [21]. In electrochemical aptamer sensors, aptamers are used for the specific recognition of target molecules, triggering changes in electrochemical signals after binding with the target and enabling determination [22]. Traditional electrochemical sensors often face a large number of challenges in terms of sensitivity and selectivity. To overcome these limitations, novel electrode materials and sensor platforms are urgently needed [23]. Two-dimensional transition metal carbides or nitrides, known as MXenes, have garnered significant interest due to their unique electronic, chemical, and mechanical properties [24]. Due to the covalent bonding between transition metals and carbon or nitrogen atoms, MXene exhibits appropriate electrical conductivity [25]. Meanwhile, MXene presents a large surface area and the ability to functionalize with various chemical groups, making them ideal candidates for biosensor applications [26,27]. Notably, MXene can be coupled with other nanomaterials and used as a modification material for ME to achieve higher sensitivity of electrochemical biosensors through a synergistic effect [28,29,30]. Recently, MXene and gold nanoparticles (AuNPs) have been extensively studied in electrochemical sensors due to their exceptional electrochemical properties and good biocompatibility [31,32].

**Figure 1:**
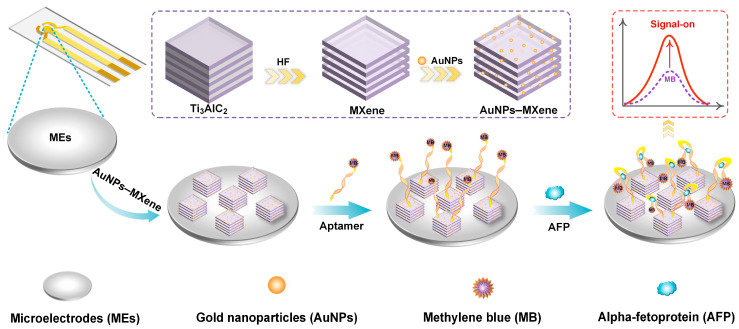
Schematic diagram of signal-on microelectrode electrochemical based on AuNPs–MXene for alpha-fetoprotein determination.

In this study, a signal-on electrochemical aptamer sensor based on AuNPs–MXene composite materials was employed to develop a sensitive AFP assay. The AuNPs–MXene composite was synthesized through a straightforward one-step method, which provided an excellent surface area with biocompatibility for ME. Due to the high selectivity of the aptamers, the sensor can identify AFP levels in complex samples. Meanwhile, as a sensitive signaling molecule, the MB signal is amplified with the increase in AFP concentration when the AFP and aptamer undergo conformational changes (Figure 1). The designed aptamer sensor demonstrates high selectivity and sensitivity and is suitable for AFP quantitation in real samples.

## 2. Materials and Methods

### 2.1. Materials and Instruments

AFP, bovine serum albumin (BSA), prostate-specific antigen (PSA), immunoglobulin G (IgG), hemoglobin (HGB), and ovalbumin (OVA) were purchased from Aladdin Reagent Co., Ltd. (Shanghai, China). Chloroauric acid hydrate (HAuCl_4_·3H_2_O), potassium ferricyanide (K_3_[Fe(CN)_6_]), potassium ferrocyanide trihydrate (K_4_[Fe(CN)_6_]·3H_2_O), Nafion, hydrofluoric acid (HF), H_2_SO_4_, phosphate-buffered saline (PBS, 0.01 M), and MB were obtained from Sigma-Aldrich Co., Ltd. (Shanghai, China). Ti_3_AlC_2_ powder was provided by XFNANO Materials Tech Co., Ltd. (Nanjing, China). The other reagents were analytically pure, not further processed, and purchased from Mackin (Shanghai, China). AFP-aptamer was obtained from Sangon biotech Co., Ltd. (Shanghai, China), with the sequences 5′-SH-GTGACGCTCCTAACGCTGACTCAGGTGCAGTTCTCGACTCGGTCTTGATGTGGGTCCTGTCCGTCCGAACCAATC-MB-3′. Ultrapure water (18.2 MΩ cm) was used in all the experiments.

A scanning electron microscope (SEM, 7800F, JEOL, Tokyo, Japan), a transmission electron microscope (TEM, 2100 Plus, JEOL, Tokyo, Japan), an X-ray diffractometer (XRD, D8 ADVANCE, Bruker, Karlsruhe, Germany), and an X-ray photoelectron spectrometer (XPS, K-Alpha, Thermo, Waltham, MA, USA) were adopted to observe the microscopic morphology of Ti_3_AlC_2_, MXene, or AuNPs–MXene. All electrochemical procedures were performed on an electrochemical workstation (CHI660, Shanghai Chenhua, Shanghai, China). Cyclic voltammetry (CV) and electrochemical impedance spectroscopy (EIS) were used to verify the modification process of the electrode, which were performed in 5 mM [Fe(CN)_6_]^3−/4−^ and 0.1 M KCl. In CV, the potential window was set as −0.2–0.6 V. In EIS, the frequency range was set as 10^−2^–10^5^ Hz. AFP determination was performed via differential pulse voltammetry (DPV).

### 2.2. Manufacture of Microelectrode

The ME was prepared via micro-electro-mechanicals system (MEMS) processes [33]. Briefly, the glass base was immersed in an acidic solution to remove surface impurities. Then, the cleaned glass sheet was put into an electron beam evaporation coating machine; after vacuuming, the 50 mm Ti layer was first steamed; and the 1000 mm Au layer was steamed. Finally, the fabricated device was placed at 300 °C in an oven for heat treatment, which improved the adhesion between the gold electrode and the substrate. Then, a single mask lithography process was used to map the structure and develop the device. In short, a double layer of negative photoresist was spun onto the substrate and mask, and, after the photolithography, the electrode pattern of the photoresist formed on the surface of the chrome-gold metal film. For the reference electrode, the shadow mask technique was used to fabricate Ag/AgCl films. Before use, the ME was sealed and stored in constant-temperature (20 °C) and -humidity (40% RH) conditions. In particular, the diameter of the working electrode was 1 mm (Supplementary Figure S1).

### 2.3. Preparation of MXene and AuNPs–MXene

MXene was formed via the aqueous acid etching method, in which the Al was stripped from Ti_3_AlC_2_ [34]. Firstly, 2 mg Ti_3_AlC_2_ was added to 20 mL of HF (45%) under stirring conditions and reacted at room temperature for 24 h. After that, the Al was removed from Ti_3_AlC_2_. Afterward, the product was centrifuged with deionized water until the solution was neutral. Finally, the resulting solid product was dissolved in 10 mL of deionized water and stored at 4 °C until further use.

AuNP–MXene composites were prepared by a chemical reduction method. Briefly, 1 mL of HAuCl_4_ (10 mM) and 1 mL of MXene dispersion were mixed under stirring conditions and maintained for 10 min. Because of the desirable self-reduction ability of MXene, AuNPs, which had no need for an additional reducing agent, were in situ grown on the MXene surface. Finally, the mixture solid was centrifuged and vacuum-dried (60 °C) to obtain AuNPs–MXene.

### 2.4. Modification of the Electrode

Before use, the electrode was activated in 0.5 M H_2_SO_4_ for 5 min in order to remove surface impurities. Then, 2 mg AuNPs–MXene was added to a binder, Nafion (5 wt%), and uniformly mixed. Subsequently, 10 μL of the obtained AuNPs–MXene was added to the ME and dried at room temperature for 30 min. Then, 2 μM of AFP aptamer was dropped on the AuNPs–MXene/ME and incubated for 1 h, in which the aptamer had a precoupled signal molecule MB. In that time, the AFP aptamer self-assembled well with AuNPs in AuNPs–MXene due to the presence of -SH bonds. Finally, 100 ng/mL AFP was added to aptamer/AuNPs–MXene/ME for incubation for 30 min and then prepared for further electrochemical analysis. Upon incubation of AFP with the electrode surface, conformational changes occurred in the aptamer–target complex, bringing MB molecules closer to the electrode surface and amplifying the signal. An intriguing signal-on mechanism was formed.

### 2.5. Preparation of Serum Samples

To verify the actual analytical performance of the prepared sensor, human blood samples were collected from Sir Run Run Shaw Hospital, affiliated with Zhejiang University. Then, the blood samples were left at room temperature for 1 h and centrifuged (4000 rpm, 20 min) to collect serum for further electrochemical analysis.

## 3. Results and Discussion

### 3.1. Characterization of Ti_3_AlC_2_, MXene, and AuNPs–MXene

**Figure 2:**
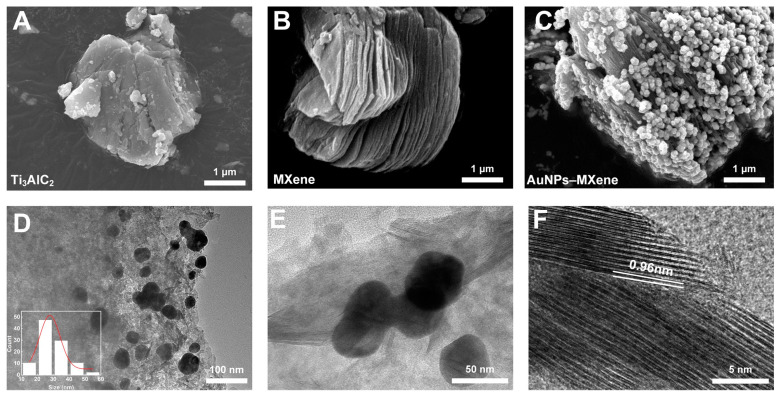
SEM images of (**A**) Ti_3_AlC_2_, (**B**) MXene, and (**C**) AuNPs–MXene. TEM image of AuNPs–MXene (**D**) and at higher magnifications (**E**). (**F**) TEM interlayer spacings of MXene.

SEM was adopted to observe the microscopic morphology of Ti_3_AlC_2_, MXene, and AuNPs–MXene. As shown in Figure 2A, the original Ti_3_AlC_2_ had a blocky structure. After HF corrosion, the Al was successfully removed from Ti_3_AlC_2_, so the MXene presented an interesting accordion structure with a high number of layers (Figure 2B). When HAuCl_4_ was subsequently added, a large number of AuNPs grew on the surface and layers of MXene (Figure 2C). In order to observe the microstructure of AuNPs–MXene, TEM with greater magnification was adopted. As described in Figure 2D, the AuNPs were evenly distributed in the MXene, indicating that the AuNPs were successfully reduced, and the average diameter was about 30 nm. Meanwhile, a higher magnification of the AuNPs is shown in Figure 2E. As illustrated in Figure 2F, the interlayer gap of MXene was about 0.96 nm [35]. In addition, the XRD and XPS patterns of the MXene were further investigated. As shown in the Supplementary Figure S2, MXene presented typical crystal faces at 002, 101, 104, 105, and 110, indicating the successful preparation of MXene [36]. Meanwhile, the XPS images of the MXene also exhibited typical F, O, Ti, and C (Supplementary Figure S3) [37]. The above results supported the successful synthesis of MXene and AuNPs–MXene.

### 3.2. Electrochemical Behavior During Gradual Modification

**Figure 3:**
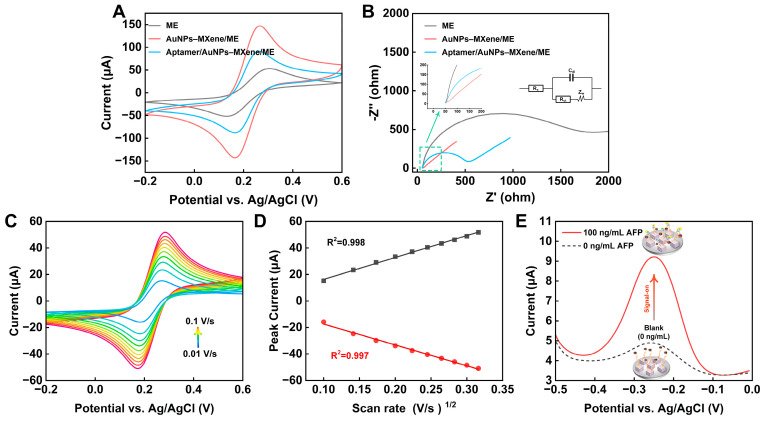
(**A**) CV and (**B**) EIS curves for stepwise modifications of bare ME, AuNPs–MXene/ME, and aptamer/AuNPs–MXene/ME in 5 mM [Fe(CN)_6_]^3−/4−^ and 0.1 M KCl. (**C**) CV and (**D**) linear curves with increasing scan rates (0.01~0.1 V/s) and peak current of ME in 5 mM [Fe(CN)_6_]^3−/4−^ and 0.1 M KCl. (**E**) DPV responses for initial 0 ng/mL and 100 ng/mL AFP incubation.

Normally, CV and EIS are used to verify the modification process of MEs, AuNPs–MXene/ME, and aptamer/AuNPs–MXene/ME. As shown in Figure 3A, the bare ME presented the typical pair of symmetrical redox peaks, and the peak-to-peak separation was calculated as about 161.1 mV. With the modification of AuNPs–MXene, the peak current showed an upward tendency. A possible reason was that the AuNP–MXene composite presented appropriate electrical conductivity and promoted the transfer of electrons [38]. However, with the modification of the AFP aptamer, the current showed a downward trend, which was because the nucleotides along the DNA strand blocked the transfer of electrons. For EIS, the semicircle diameter in the Nyquist diagram is equivalent to the charge transfer resistance (R_ct_), which is closely related to the charge transfer kinetics of the redox probe on the electrode interface [39]. The general Randle equivalent circuit is shown in the inset in Figure 3B, in which the R_S_, C_dl_, R_ct_, and Z_w_ represent the solution resistance, double-layer capacitance, electron-transfer resistance, and Warburg diffusion resistance. In Figure 3B, a larger R_ct_ can be observed for the bare ME. After AuNPs–MXene modification, the R_ct_ decreased significantly, indicating that the AuNPs–MXene promoted the electron transfer of the redox probe. Subsequently, the R_ct_ became larger with aptamer incubation, which was attributed to the presence of DNA strands on the electrode surface. Therefore, the above CV and EIS results suggested the successful modification of the aptamer/AuNPs–MXene/ME. To verify the diffusion efficiency of the developed ME, the bare ME was tested with increasing scan rates (0.01~0.1 V/s). As shown in Figure 3C, the peak current increased as the scan rates increased. Particularly, a linear relationship was well established between the peak current and the square root of the scan rates, which perfectly conformed to the Randles–Sevčik equation [40] (Figure 3D).

Furthermore, to evaluate the signal-on mechanism, 0 ng/mL and 100 ng/mL AFP were separately incubated with the aptamer/AuNPs–MXene/ME. Primarily, the MB presented a significant peak due to the presence of MB molecules on the aptamer, without AFP incubation. Then, 100 ng/mL AFP was incubated; an increased MB signal could be clearly observed (Figure 3E). A reasonable explanation for this is that the AFP was specifically recognized by AFP aptamer, so the MB molecules became closer to the electrode surface and amplified the signal.

### 3.3. Optimization of Experimental Parameters

**Figure 4:**
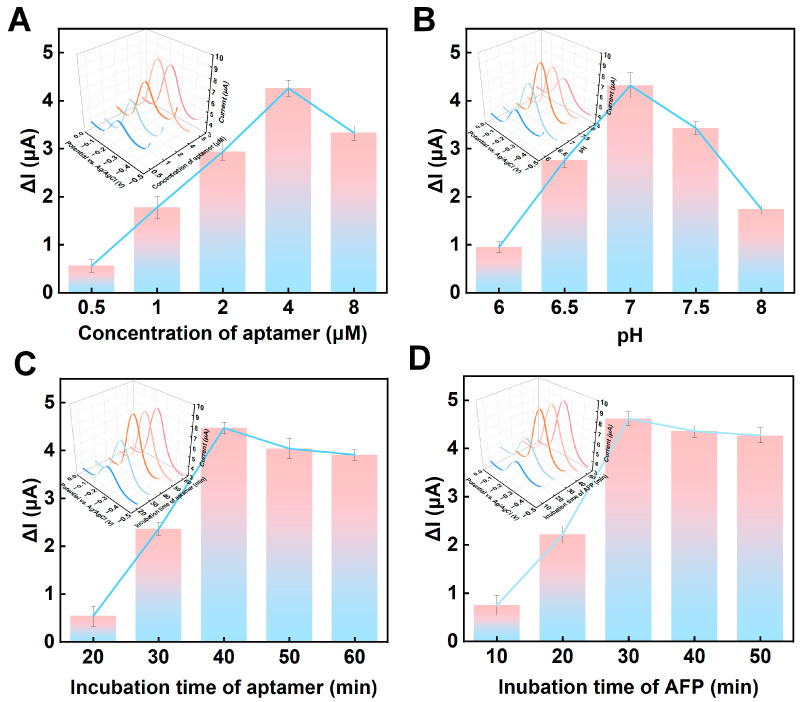
Optimizations of (**A**) concentration of the aptamer, (**B**) pH, (**C**) incubation time of the aptamer, and (**D**) incubation time of the AFP.

To efficiently and sensitively detect AFP, key experimental parameters, including the concentration of the AFP aptamer, pH of the buffer, aptamer incubation time, and AFP incubation time were optimized. For each optimization condition, the signals before and after AFP incubation were explored with voltammetry analysis. Due to the combination between the aptamer and target, the MB signal was increased from the original 4.9 μA. Firstly, the concentration of the AFP aptamer was optimized, where the concentration level was set to 0.5–8, and the current change value (ΔI) before and after the addition of 100 ng/mL AFP was recorded. In Figure 4A, ΔI shows an upward tendency as the AFP aptamer concentration gradually increases from 0.5 μM. When the concentration reached 4 μM, ΔI reached the maximum. A possible reason for this is the complete reaction between the AFP aptamer and the target object AFP, while excessive aptamer could hinder electron transfer on the electrode surface [41]. Therefore, 4 μM was chosen as the optimal AFP aptamer concentration. Similarly, since the activity of proteins can be affected by the pH, the pH of the buffer was optimized (Figure 4B). As the pH gradually increased from 6 to 7, the ΔI generally increased; when the pH exceeded 7, the ΔI showed the opposite trend. Since neutral conditions were the most suitable environmental conditions for the aptamer, pH 7 was used as the optimal pH condition [42]. Subsequently, the incubation time of the AFP aptamers was optimized (Figure 4C). When the incubation time of the AFP aptamers was increased from 20 min to 40 min, the ΔI raised due to the SH- and AuNPs in the AuNPs–MXene forming Au-S on the electrode surface. When the incubation time exceeded 40 min, the ΔI did not change considerably owing to aptamer saturation. Therefore, 40 min was set as the optimal incubation time for the AFP aptamers. Finally, the incubation time of the AFP was optimized (Figure 4D). With the increase in the incubation time, the ΔI presented an uptrend until 30 min. After 30 min, the ΔI remained unchanged, which was due to the AFP aptamers and AFP being fully integrated and saturated [43].

### 3.4. Analytical Performance

**Figure 5:**
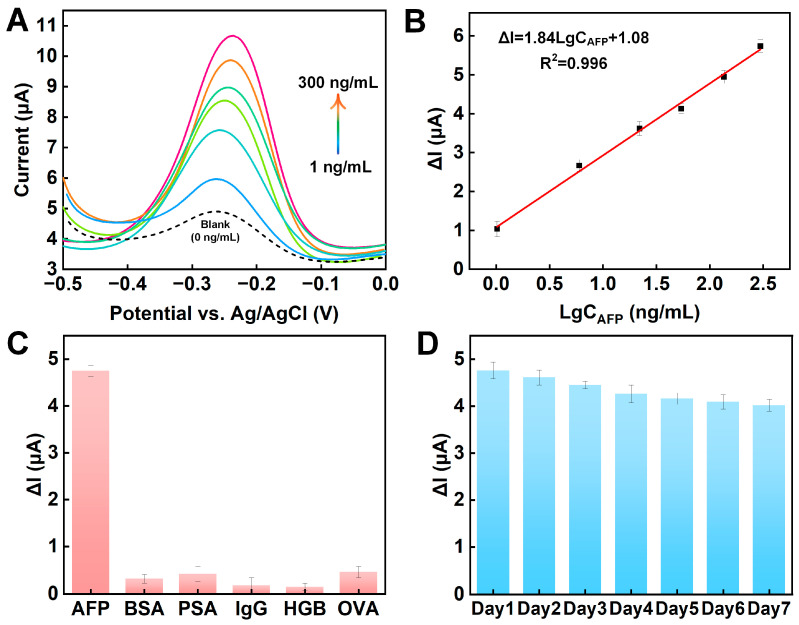
(**A**) DPV and (**B**) log-linear calibration plots for increasing levels of AFP. (**C**) Selectivity and (**D**) long-term stability of proposed sensor.

Under the optimal experimental conditions, different levels of AFP were measured via DPV using the prepared sensor. As described in Figure 5A, without AFP, the aptamer/AuNPs–MXene/ME exhibited a certain peak current, which was due to the electrical signal generated by the signal molecule MB on the aptamer (blank line). With increasing AFP levels, the peak current of MB showed a rising tendency. A reasonable explanation is that the AFP binds to the AFP aptamer, resulting in the folding of the aptamer chain. At this time, the distance between the MB and the electrode surface is shortened, so the peak current of MB is reasonably increased. In the range of 1–300 ng/mL AFP, we found no linear relationship between C_AFP_ and ΔI (Supplementary Figure S4), so the logarithm of C_AFP_ with the ΔI was established (Figure 5B). A linear curve was well defined as ΔI = 1.84LgC_AFP_ + 1.08 (R^2^ = 0.996), and the limit of determination (LOD) (S/N = 3) and limit of quantification (LOQ) (S/N = 10) were, respectively, calculated as 0.05 and 0.167 ng/mL. In addition, the characteristics of this method and other methods in the literature are summarized in Supplementary Table S1.

### 3.5. Selectivity, Reproducibility, and Stability

During real measurements, large numbers of biomolecules, such as BAS, PSA, IgG, HGB, and OVA, can interfere with the determination of AFP. Therefore, the selectivity of the manufactured aptamer/AuNPs–MXene/ME sensor was explored. As shown in Figure 5C, the ΔI value of the 100 ng/mL AFP was steady. However, when a 100 times larger concentration of the interfering molecules (BAS, PSA, IgG, HGB, or OVA) was used, a weak ΔI was observed. The results showed that the aptamer/AuNPs–MXene/ME electrode presented satisfactory selectivity for AFP, owing to the specificity between the aptamer and the target.

Subsequently, the reproducibility of the fabricated aptamer/AuNPs–MXene/ME sensor was investigated. Under the same experimental conditions, five electrodes were made for AFP determination. The ΔI values for all the electrodes were similar, with the relative standard deviation calculated as 5.7% (Supplementary Figure S5). The results showed that the proposed sensor presented excellent reproducibility. Finally, the long-term stability of the prepared aptamer/AuNPs–MXene/ME sensor was studied. The sensors were stored at 4 °C, and the electrochemical signal was measured every day for seven days. As depicted in Figure 5D, the signal fluctuation of the ΔI within seven days was well controlled to less than 8.4%, indicating that the sensor presented acceptable long-term stability. In addition, the stability of the proposed signaling molecule MB was investigated (Supplementary Figure S6). Over 20 CV cycles of −0.5–0.2 V, the aptamer/AuNP–MXene/ME signals overlapped and exhibited satisfactory structural stability.

### 3.6. Real Sample Analysis

**Figure 6:**
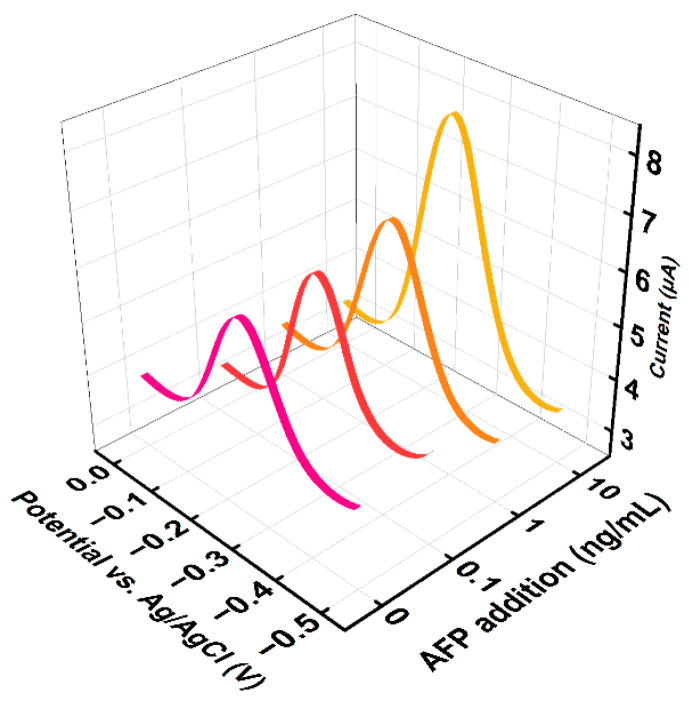
DPVs responses of original 0 (crimson) ng/mL AFP sample and 0.1 (red), 1 (orange), or 10 (yellow) ng/mL AFP.

In order to explore the real analytical capability of the sensor, the analytical capability of the proposed aptamer/AuNPs–MXene/ME sensor was evaluated using real human serum samples. Firstly, a DPV test was performed on the collected serum samples to calculate the original AFP level. As shown in Figure 6, the DPV signal of the original AFP level was about 6.15 μA, and the ΔI was determined as 1.26. According to the linear curve ΔI = 1.84LgC_AFP_ + 1.08, the original level of the AFP was calculated as about 1.25 ng/mL. Then, 0.1, 1, or 10 ng/mL AFP was added to the sample. Finally, the AFP concentration and recovery rate using real human serum samples were carefully calculated (Figure 6). As shown in Table 1, the proposed sensor had a satisfactory recovery rate with AFP addition, suggesting the aptamer/AuNPs–MXene/ME sensor can be used as a practical tool for the clinical determination of AFP.

## 4. Conclusions

In conclusion, a novel signal-on electrochemical aptamer sensor, utilizing AuNP–MXene composite materials, was successfully developed for AFP determination. The one-step synthesis of the AuNP–MXene composite enabled the production of a highly effective sensing platform, which offers significant improvements in sensitivity and selectivity. The self-assembly of the aptamers allows for the specific recognition of AFP, while the signal molecule, MB, facilitates the real-time monitoring of the conformational changes within the aptamer–target complex. The interaction results in a signal-on electrochemical signal, indicating the alterations in the levels of AFP. The sensor exhibits high sensitivity and selectivity in AFP determination and shows promise for application in real sample analyses. Currently, the portability and real-time analysis capabilities of the proposed sensor are some of its limitations. In the future, the efforts to integrate the proposed sensor into portable diagnostic devices are expected to enable the effective in situ monitoring of AFP levels.

## Figures and Tables

**Table 1:** Analyses of AFP in natural human serum samples (ng/mL). In the recovery test, the concentration of the AFP was 0.1, 1, or 10 ng/mL.

Species		Original Level	Added	Found	Recovery
Human serum	Sample 1	1.25	0.1	1.37	101.5%
	Sample 2	1.25	1	2.22	98.7%
	Sample 3	1.25	10	11.65	103.6%

## Data Availability

The data that support the findings of this study are available from the corresponding author upon reasonable request.

## Supplementary Materials

The following supporting information can be downloaded at: https://www.mdpi.com/article/10.3390/s24247878/s1. Figure S1. Batch manufacturing and individual microelectrode. The working electrode, reference electrode, and the counter electrode were appropriately manufactured. Figure S2. XRD image of MXene. The typical crystal faces of (002), (101), (104), (105), (110) appeared in the MXene. Figure S3. XPS image of MXene. The typical elements of F (21.37%), O (19.38%), Ti (19.77%), and C (39.48%) appeared in the MXene. Figure S4. The original concentration of AFP within the ΔI. The AFP C_AFP_ does not show a good linear relationship with ΔI. Figure S5. Reproducibility exploration of the proposed sensor. The ΔI of the five electrodes exhibited similar values, and the relative standard deviation was calculated as 5.7%. Figure S6. Stability study of MB via repetitive CV. MB signals exhibited satisfactory structural stability. Table S1. Summary of this work and the previous remarkable literature on electrochemistry sensors for AFP detection. References [43,44,45] are cited in the supplementary materials.
