# Supplementary material for: A Signal-On Microelectrode Electrochemical Aptamer Sensor Based on AuNPs–MXene for Alpha-Fetoprotein Determination
Source: Sensors (Basel). 2024 Dec 10;24(24):7878. doi: 10.3390/s24247878 (PMC11678932; doi:10.3390/s24247878)
Supplement: Supplementary file 1 [file sensors-24-07878-s001.zip › sensors-3284676-supplementary.pdf]

Supplementary Material for

A signal-on microelectrode electrochemical aptamer sensor based on AuNPs–MXene for alpha-fetoprotein determination

Xiaoyu Su [1,†], Junbiao Chen [2,†], Shanshan Wu [2], Yong Qiu [3,4,*] and Yuxiang Pan [1,*]

[1] Innovation Platform of Micro/Nano Technology for Biosensing, ZJU-Hangzhou Global Scientific and Technological Innovation Center, Zhejiang University, Hangzhou 311200, China; xiaoyusu@zju.edu.cn

[2] Hangzhou Shuntai Installation Engineering Co., Ltd., Hangzhou 311200, China; cjb125125@126.com (J.C.); wuss0926@126.com (S.W.)

[3] Biosensor National Special Laboratory, Department of Biomedical Engineering, Zhejiang University, Hangzhou 310027, China

[4] Binjiang Institute, Zhejiang University, Hangzhou 310053, China

[*] Correspondence: zjubme_qy@zju.edu.cn (Y.Q.); panyuxiang@zju.edu.cn (Y.P.)

[†] These authors contributed equally to this work.

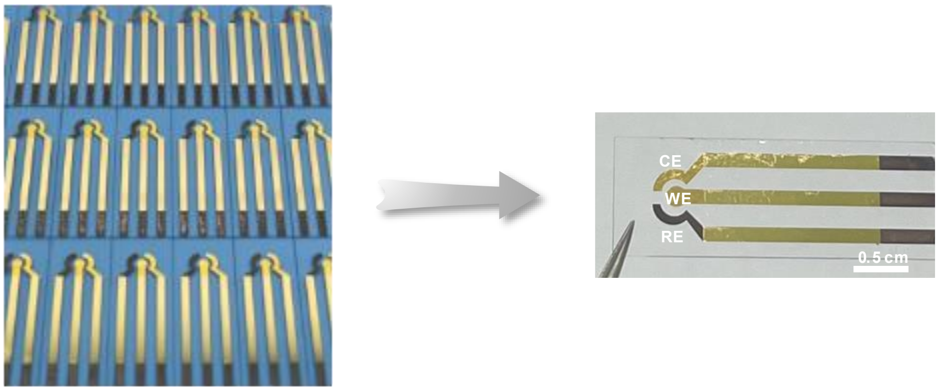

Figure. S1. Batch manufacturing and individual microelectrode. The working electrode, reference electrode, and the counter electrode were appropriately manufactured.

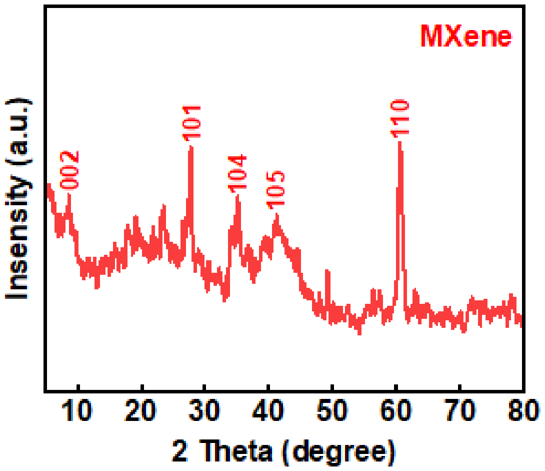

Figure. S2. XRD image of MXene. The typical crystal faces of (002), (101), (104), (105), (110) appeared in MXene.

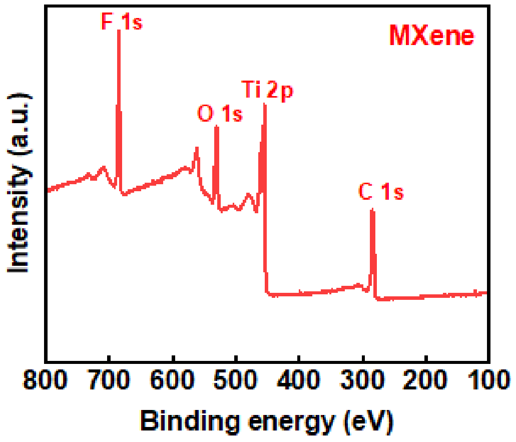

Figure. S3. XPS image of MXene. The typical elements of F (21.37%), O (19.38%), Ti (19.77%), and C (39.48%) appeared in MXene.

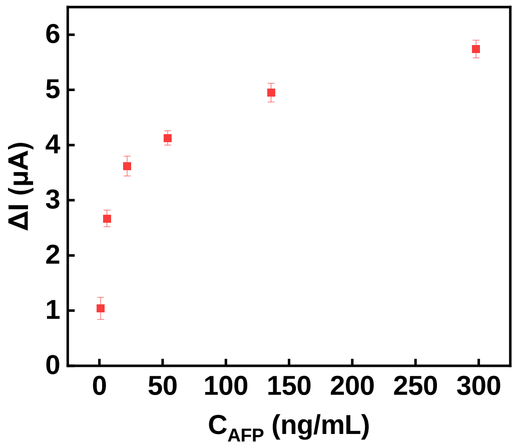

Figure S4. The original concentration of AFP within the ΔI. The AFP CAFP does not show a good linear relationship with ΔI.

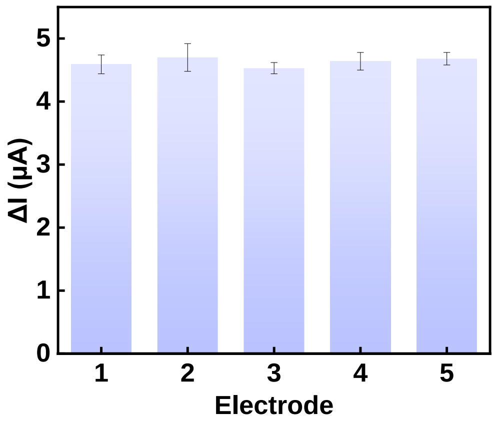

Figure S5. Reproducibility exploration of proposed sensor. The ΔI of five electrodes exhibited the similar value, and the relative standard deviation was calculated as 5.7%.

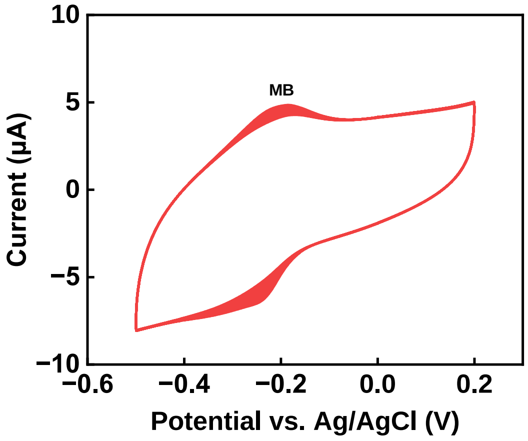

Figure. S6. Stability study of MB via repetitive CV. MB signals exhibited satisfactory structural stability

Table S1. Summarization of this work and previous remarkable literature on electrochemistry sensor for AFP detection.

Material	Linear range (ng/mL)	LOD (ng/mL)	Reference
anti–AFP/AuNPs/ PGNR	5–60	1	[1]
TH/RGO/Au NPs	100–105	50	[2]
Fe3O4-ε-PL-Hep NPs	0.1 to 100	0.072	[3]
aptamer/MXene–Au/SPE	1-300	0.05	This work

References

1. Jothi, L.; Jaganathan, S. K.; Nageswaran, G., An electrodeposited Au nanoparticle/porous graphene nanoribbon composite for electrochemical detection of alpha-fetoprotein. *Materials Chemistry and Physics* **2020,** 242, 122514.
2. Li, G.; Li, S.; Wang, Z.; Xue, Y.; Dong, C.; Zeng, J.; Huang, Y.; Liang, J.; Zhou, Z., Label-free electrochemical aptasensor for detection of alpha-fetoprotein based on AFP-aptamer and thionin/reduced graphene oxide/gold nanoparticles. *Analytical Biochemistry* **2018,** 547, 37-44.
3. Xu, T.; Chi, B.; Wu, F.; Ma, S.; Zhan, S.; Yi, M.; Xu, H.; Mao, C., A sensitive label-free immunosensor for detection α-Fetoprotein in whole blood based on anticoagulating magnetic nanoparticles. *Biosensors and Bioelectronics* **2017,** 95, 87-93.
